# Bacterial Urinary Tract Infection and Early Asymptomatic Bacteriuria in Kidney Transplantation Still Negatively Affect Kidney Transplant Outcomes in the Era of Modern Immunosuppression and Cotrimoxazole Prophylaxis

**DOI:** 10.3390/biomedicines10112984

**Published:** 2022-11-20

**Authors:** Chayanan Santithanmakorn, Jakapat Vanichanan, Natavudh Townamchai, Kamonwan Jutivorakool, Salin Wattanatorn, Methee Sutherasan, Julin Opanuruk, Stephen J. Kerr, Kearkiat Praditpornsilpa, Yingyos Avihingsanon, Suwasin Udomkarnjananun

**Affiliations:** 1 Department of Medicine, Faculty of Medicine, Chulalongkorn University and King Chulalongkorn Memorial Hospital, Bangkok 10330, Thailand; 2 Division of Infectious Diseases, Department of Medicine, Faculty of Medicine, Chulalongkorn University and King Chulalongkorn Memorial Hospital, Bangkok 10330, Thailand; 3 Division of Nephrology, Department of Medicine, Faculty of Medicine, Chulalongkorn University and King Chulalongkorn Memorial Hospital, Bangkok 10330, Thailand; 4 Excellence Center for Organ Transplantation (ECOT), King Chulalongkorn Memorial Hospital, Thai Red Cross Society, Bangkok 10330, Thailand; 5 Renal Immunology and Transplantation Research Unit, Faculty of Medicine, Chulalongkorn University, Bangkok 10330, Thailand; 6 Department of Surgery, Faculty of Medicine, Chulalongkorn University and King Chulalongkorn Memorial Hospital, Bangkok 10330, Thailand; 7 Biostatistics Excellence Centre, Research Affairs, Faculty of Medicine, Chulalongkorn University, Bangkok 10330, Thailand; 8 HIV-NAT, Thai Red Cross AIDS Research Centre, Bangkok 10330, Thailand; 9 The Kirby Institute, University of New South Wales, Sydney 2052, Australia

**Keywords:** asymptomatic bacteriuria, immunosuppression, kidney transplantation, overweight, tacrolimus, urinary tract infection

## Abstract

Risk factors and consequences of urinary tract infection (UTI) post-kidney transplant have been variously reported by studies that were heterogenous in immunosuppressants and prophylactic protocols. We aimed to clarify the risks and consequences of UTI in kidney transplant recipients with post-transplantation cotrimoxazole prophylaxis in the context of modern immunosuppression. This retrospective cohort included kidney transplant recipients receiving tacrolimus, mycophenolate, prednisolone, and cotrimoxazole for bacterial UTI prophylaxis. Recipients were categorized into non-UTI and UTI groups. Asymptomatic bacteriuria (ASB) was screened in the first 3 months and was evaluated for association with UTI. Of 348 kidney transplant recipients, 129 were in the UTI group and 219 in the non-UTI group. UTI risk factors were female sex, body mass index ≥ 25 kg/m^2^, human leukocyte antigen mismatch, and panel reactive antibody ≥ 50%. Recipients with recurrent UTI had inferior allograft function compared with non-UTI recipients. Patient survival was significantly lower in recipients with UTI in the first post-transplant month. Higher degree of immunosuppressions was associated with recurrent UTI and drug-resistant organisms. In conclusion, UTI continues to negatively affect graft function and survival of kidney transplant recipients. Treating ASB in the first 3 months did not reduce the UTI incidence in the first transplantation year.

## 1. Introduction

Urinary tract infection (UTI) is the most common infection after kidney transplantation [1,2,3], and kidney transplant recipients have a higher risk of UTI compared with the general population [4,5]. UTI prevalence among kidney transplant recipients ranges from 20 to 80% due to differences in definitions, center-specific preventive strategies, and kidney transplant characteristics [3,6,7,8].

Several transplant-related factors are associated with UTI after kidney transplantation, including the effect of immunosuppression, bladder catheterization and ureteral stents, deceased donor transplantation, and acute rejection episodes [7,9,10]. However, the effect of bacterial UTI on kidney transplant outcomes has not been clearly established. Previous studies have shown conflicting results regarding the impact of UTI on patient and kidney allograft survival [11,12,13,14]. The interpretation of these study results is confounded by the use of different immunosuppression protocols and UTI prevention strategies. The risks and effects of bacterial UTI in the current era of the triple immunosuppressive medications strategy including tacrolimus, mycophenolic acid (MPA), and corticosteroids, along with the use of cotrimoxazole for bacterial UTI prophylaxis have yet to be definitively determined [6,7,15,16].

Another uncertain aspect regarding UTI after kidney transplantation relates to the treatment of asymptomatic bacteriuria (ASB) to prevent UTI [17,18]. Data regarding the treatment of ASB in the first 2 to 3 months after kidney transplantation is still limited, although a recent multicenter randomized controlled pragmatic trial (RCT) reported that the ‘screen-and-treat’ strategy for ASB after 2 months of transplantation did not reduce the occurrence of UTI [19]. The American Society of Transplantation Infectious Diseases Community of Practice suggests that 5-day antibiotic treatment can be considered for ASB within the first 2 months, while acknowledging that the benefit is uncertain and may increase the risk of drug-resistant organisms [3]. Sebe et al. demonstrated that the treatment of ASB during the first transplant year increased the risk of drug-resistant organisms [17].

The aims of this study were to more closely examine these unresolved issues surrounding UTI in kidney transplantation in recipients receiving standard tacrolimus-MPA-corticosteroid immunosuppression, which has the best evidence for prevention of transplant rejection compared with other regimens [20,21], and cotrimoxazole, which is the current suggested prophylaxis for post-transplant *Pneumocystis jirovecii* pneumonia (PCP) and bacterial UTI [3,22]. Our primary aim was to evaluate risk factors associated with bacterial UTI and assess the effects on patient and allograft survival and kidney allograft function. As a secondary outcome, we explored the incidence of ASB in the first 90 days after transplantation and its associated factors and consequences. 

## 2. Methods

### 2.1. Study Design and Study Population

This retrospective cohort study was conducted in adult kidney transplant recipients transplanted at King Chulalongkorn Memorial Hospital, a tertiary transplant center in Bangkok, Thailand, from 2010 to 2019. Inclusion criteria were age ≥ 18 years at the time of transplant and receiving tacrolimus/MPA/corticosteroid immunosuppression. Our institutional protocol for maintenance immunosuppression consisted of tacrolimus dosed once or twice daily (targeting pre-dose concentration 7–10 ng/mL in the first 3 months and 4–7 ng/mL thereafter), MPA (either mycophenolate mofetil—MMF—or enteric-coated mycophenolate sodium—MPS), and prednisolone. The induction immunosuppression was either basiliximab or anti-thymocyte globulin. In accordance with institutional protocols, all patients received 1 g of Ceftriaxone intravenously at the time of surgery for perioperative infection prophylaxis unless a history of major allergic reaction had been documented and standard bacterial UTI and PCP prophylaxis with daily cotrimoxazole (160 mg trimethoprim and 800 mg sulfamethoxazole) for at least 12 months [4,15,23]. Cotrimoxazole prophylaxis of PCP was continued during the treatment of UTI or other infections. Patients not treated with cotrimoxazole within the first year were excluded from this study. Screening for bacteriuria occurred weekly until hospital discharge, then every 2–4 weeks until 3 months after transplantation. The treatment of ASB was based on the treating physician’s decision, with antibiotics selection according to antibiogram, and given for 5–7 days. According to our institutional protocol, the urinary bladder catheter (Foley catheter) was removed on day 7 after transplantation, the surgical drainage tube was removed on day 8, and the ureteral stent was removed after discharge in the 4th–5th week post transplantation at the out-patient clinic. These standard protocols were adjusted according to individual patient conditions. 

### 2.2. Outcomes and Definitions

Recipients were categorized into UTI and non-UTI groups. The UTI group included kidney transplant recipients with at least one episode of bacterial UTI during the follow-up period; recipients who never experienced UTI were classified as non-UTI. The primary study objectives were to examine risk factors for bacterial UTI after kidney transplantation and to assess how bacterial UTI affected kidney transplant outcomes. The effects of UTI on kidney transplant outcomes are presented in association with patient survival, allograft survival, and estimated glomerular filtration rate (eGFR) of kidney allograft using the Chronic Kidney Disease Epidemiology Collaboration (CKD-EPI) equation [24]. 

UTI in this study included both uncomplicated UTI and complicated UTI of bacterial origin. Recipients who experienced any UTI caused by fungi or mycobacterium were excluded from this study. Uncomplicated UTI (acute simple cystitis) was defined as presenting with lower urinary tract symptoms (dysuria, frequency, or suprapubic pain) only and a positive urine culture. Recipients with complicated UTI or acute allograft pyelonephritis additionally had systemic symptoms such as fever, malaise, or bacteremia with the same organism as cultured in urine. However, it should be mentioned that clear discrimination between simple cystitis and (mild) acute pyelonephritis is sometimes difficult, since symptoms might be masked from immunosuppressive medications that suppress inflammation and by surgical denervation of the kidney allograft [7,25]. In recipients with negative urine cultures, a UTI diagnosis was made if there were no other sources of infection in the presence of urinary tract symptoms. The colony-forming unit/mL cutoff for the diagnosis of UTI was in accordance with standard guidelines [3]. Contaminated urine cultures were considered negative and repeat urine cultures were obtained.

For kidney transplant recipients in the UTI group, the information of immunosuppression at the time of diagnosis was evaluated for the risk of having multi-drug resistant (MDR) organisms defined using non-susceptible to ≥3 antimicrobial categories and recurrent UTI defined as the occurrence of ≥3 UTI in 12 months or ≥2 UTI in 6 months [7,26,27]. ASB is defined via the positive bacterial culture from urine screening protocol in the absence of signs and symptoms of inflammation. The progression from ASB to UTI was analyzed in our cohort via survival analysis (as described in the statistical analysis).

### 2.3. Statistical Analysis

Continuous variables are presented as mean ± standard deviation (SD) or median interquartile range (IQR) according to their distribution. Categorical variables are presented as frequency (percentage). Unpaired *t*-tests, analysis of variance (ANOVA), or chi-square tests were used to make formal comparisons between UTI groups. Mortality and graft failure by UTI group were plotted using Kaplan—Meier curves, and formal comparisons were made with the Log-rank test. Thereafter, we used univariable and multivariable Cox regression to quantitate the relative risk of factors associated with UTI development. Logistic regression was performed to assess associations between potential risk factors and ASB in the first 90 days after transplantation. For recipients in the UTI group, the immunosuppressive medications at the time of UTI diagnosis were assessed for associations with the presence of MDR organisms at first UTI and recurrent UTI in those with >1 episode using logistic regression. In both logistic and Cox models, the linearity of continuous variables against the logit and hazard functions was assessed, and in the case of non-linearity, the variable was modelled in quartiles; adjacent quartiles were collapsed together if the effect sizes and 95%CI were similar; variables with *p*-values < 0.10 in univariable analysis were adjusted for in the multivariable model. The proportional hazards assumption was tested using Schoenfeld’s residuals. The fit of the logistic models was tested via the Hosmer and Lemeshow goodness of fit test. *p*-values < 0.05 were considered statistically significant. All statistical analyses were performed using Stata 17.0 (StataCorp LLC, College Station, TX, USA) and GraphPad Prism version 9.4.0 for Windows (GraphPad Software, San Diego, CA, USA).

### 2.4. Ethical Considerations

The Institutional Review Board of the Faculty of Medicine, Chulalongkorn University, Bangkok, Thailand, approved this study (IRB No. 528/63), which was conducted in compliance with the international guidelines for human research protection as described in the Declaration of Helsinki, The Belmont Report, the CIOMS Guideline, and the International Conference on Harmonization in Good Clinical Practice (ICH-GCP). Informed consent was obtained from all subjects and/or their legal guardian(s). 

## 3. Results

### 3.1. Baseline Characteristics of Kidney Transplant Recipients

Of 457 recipients, 348 were eligible for the study (Supplementary Figure S1). A total of 129/348 (37%) recipients experienced at least one episode of UTI and were categorized to the UTI group, and 219 (63%) recipients never experienced UTI and were considered as the non-UTI group. Baseline characteristics are shown in Table 1. The median follow-up time was 5.5 (3.7–7.5) years. In the UTI group, the median time to develop (first) UTI was 31 (9–283) days after transplantation, in which 118 recipients (91%) experienced complicated UTI (i.e., acute allograft pyelonephritis). Compared with the non-UTI group, recipients in the UTI group were significantly more likely to be female (59.7% vs. 31.1%, *p*-value < 0.001) and recipients of a deceased donor (61.4% vs. 50.5%, *p*-value = 0.049), had a higher proportion of panel reactive antibody (PRA) >50% (18.6% vs. 6.9%, *p*-value = 0.001), and were more likely to have a total ischemic time >12 h (56.7% vs. 45.1%, *p*-value = 0.039). Causes of kidney disease, including cystic kidney disease and obstructive/reflux nephropathy, were not different between recipients with or without UTI. Since patients followed standard protocols, the variability was low for the removal of the urinary catheter, surgical drainage tube, and ureteric stent, and there were no significant differences between the UTI and non-UTI groups. In the UTI group, 73 recipients had a single episode of UTI and 56 recipients experienced recurrent UTI. Of these 56 patients with recurrent UTI, 28 patients had a UTI within the first month after transplantation.

### 3.2. Risk Factors for Developing UTI

Hazard ratios (HR) for baseline characteristics and risk of developing UTI after kidney transplantation are shown in Table 2. Factors that were significantly associated with developing a UTI in our multivariable analysis were female sex (adjusted HR 2.25; 95%-CI 1.56–3.24; *p*-value < 0.001), body mass index (BMI) ≥25 kg/m^2^ (adjusted HR 1.57; 95%-CI 1.01–2.44; *p*-value = 0.044), increasing number of human leukocyte antigen (HLA) mismatches (adjusted HR 1.14; 95%-CI 1.01–1.29; *p*-value = 0.041), and panel reactive antibody (PRA) ≥50% (adjusted HR 1.67; 95%-CI 1.03–2.72; *p*-value = 0.038). The incidence of UTI according to BMI category is described in Supplementary Table S1.

### 3.3. Association between UTI and Kidney Transplant Outcomes

**Figure 1:**
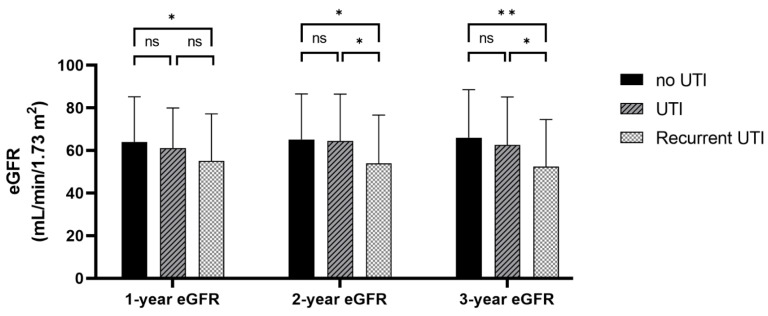
Estimated glomerular filtration rate at 1, 2, and 3 years after kidney transplantation according to the previous episode of UTI or recurrent UTI. * *p*-value < 0.05, ** *p*-value < 0.001, ns; *p*-value > 0.05.

Figure 1 illustrates the mean eGFR at 1, 2, and 3 years after transplantation according to the episodes of UTI. Recipients with recurrent UTI had a significantly lower eGFR than recipients who never had UTI at 1 year (55 ± 22 vs. 64 ± 21 mL/min/1.73 m^2^, *p*-value = 0.010), 2 years (54 ± 23 vs. 65 ± 21 mL/min/1.73 m^2^; *p*-value = 0.001), and 3 years (52 ± 22 vs. 66 ± 23 mL/min/1.73 m^2^; *p*-value <0.001). Recipients with recurrent UTI showed a significantly lower 2-year and 3-year eGFR compared with recipients with only a single episode of UTI (54 ± 23 vs. 64 ± 22 mL/min/1.73 m^2^; *p*-value = 0.013 and 52 ± 22 vs. 63 ± 22 mL/min/1.73 m^2^; *p*-value = 0.017, respectively).

**Figure 2:**
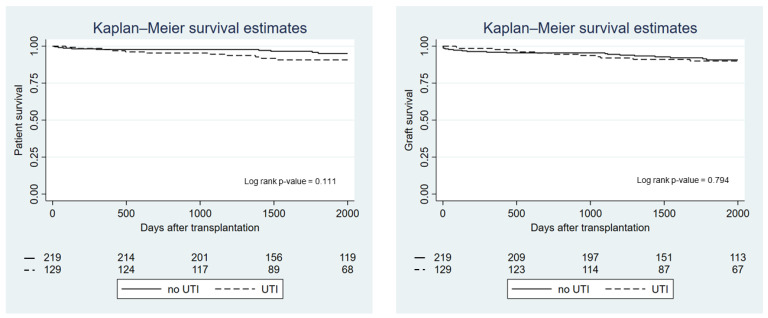
Kaplan—Meier graph showing patient and graft survival by UTI group.

**Figure 3:**
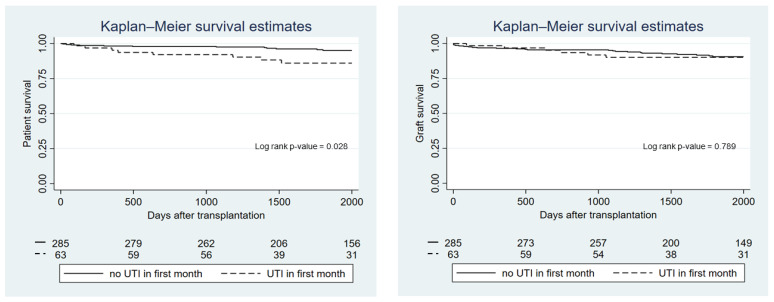
Kaplan—Meier graph showing patient and graft survival according to whether patients experienced a UTI within the first month after transplantation.

Patient and allograft survival by UTI group are presented in Figure 2. There was no difference between groups in terms of overall survival (*p*-value = 0.11) or allograft survival (*p*-value = 0.79). However, recipients who developed a UTI within the first month after transplantation had significantly lower patient survival compared with recipients who did not experience a UTI in the first month (*p*-value = 0.028), although this did not affect kidney allograft survival (*p*-value = 0.79) (Figure 3). Using Cox proportional hazard, the hazard ratio for death in recipients who had a UTI within the first month was 2.38 (95%-CI 1.07–5.31; *p*-value = 0.033). Recipients with recurrent UTI also did not show any difference in overall and allograft survival compared with the non-UTI group (*p*-value = 0.12 and *p*-value = 0.82, respectively). Rejection episodes were not different between recipients in the UTI and the non-UTI group (27.1% vs. 19.6%; *p*-value = 0.123).

### 3.4. Type of Causative Organisms

Table 3 details the urine culture result of the recipients in the UTI group at first UTI diagnosis. Of 129 kidney transplant recipients in the UTI group, 63 recipients (48%) had a UTI diagnosed in the first month after transplantation and 98 recipients (76%) experienced a UTI within the first year. *Escherichia coli* was the most frequent causative organism regardless of the timing of UTI. A total of 89 out of 129 recipients (69%) had cotrimoxazole-resistant organisms.

### 3.5. Association between Immunosuppressive Medications and UTI

Since every kidney transplant recipient in this study received the same immunosuppressive regimen (tacrolimus/MPA/prednisolone), we could compare the dosage of immunosuppressive medications at the time of UTI diagnosis for associations with MDR causative organisms or development of recurrent UTI (Table 4). In our multivariable model, tacrolimus whole blood pre-dose concentration (C_0_) and dosage of prednisolone significantly increased the odds ratio (OR) of having an MDR organism at first UTI (adjusted OR 1.20, 95%-CI 1.02–1.42; *p*-value = 0.032 and adjusted OR 1.04; 95%-CI 1.01–1.07; *p*-value = 0.007, respectively) after adjusting for tacrolimus and MPA dose. The odds of having subsequent recurrent UTI were associated with tacrolimus dosage (adjusted OR 1.26; 1.07–1.50; 95%-CI 0.007) and tacrolimus C_0_ (adjusted OR 1.28; 95%-CI 1.10–1.50; *p*-value = 0.002).

### 3.6. Risk Factors and Consequences of ASB within the First 90 Days after Transplantation

Table 5 demonstrates the OR for baseline characteristics and development of ASB within the first 90 days after kidney transplantation. Only female sex (adjusted OR 4.60; 95%-CI 2.11–10.04; *p*-value < 0.001) and increasing donor age (adjusted OR 1.05; 95%-CI 1.01–1.08; *p*-value = 0.006) were independently associated with ASB within the first 90 days from the multivariable model. Secondary analysis comparing between patients with ASB that did not progress to UTI, patients with simple cystitis, and patients with acute pyelonephritis is described in Supplementary Table S2.

**Figure 4:**
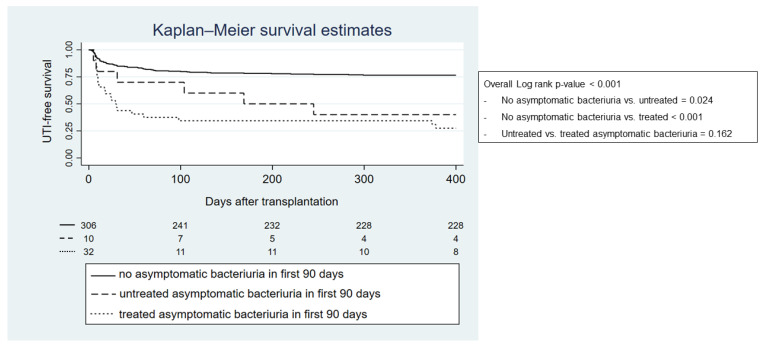
Kaplan—Meier graph showing first year UTI-free survival regarding previously treated or untreated asymptomatic bacteriuria in the first 90 days after transplantation.

From a total of 42 recipients with ASB, 32 recipients received antibiotics (76%) (cephalosporins 14 patients, quinolones 11 patients, and amoxicillin/amoxicillin-clavulanic acid 7 patients). The Kaplan—Meier curve indicates that UTI incidence in the first year after transplantation was higher in patients with ASB within the first 90 days compared with recipients who did not have ASB, regardless of whether they were treated or not (*p*-value < 0.001) (Figure 4). The hazard ratio of ASB for developing UTI in the first year was 4.01 (95%-CI 2.60–6.19; *p*-value < 0.001). Table 6 demonstrates that the incidence of UTI with an MDR causative organism in the first transplantation year was higher in recipients who were treated for ASB within the first 90 days after transplantation compared with recipients who did not receive treatment (*p*-value < 0.001).

## 4. Discussion

Our study comprehensively analyzed the risk factors and the effects of bacterial UTI in kidney transplant recipients receiving the current standard immunosuppressive regimen (tacrolimus/MPA/prednisolone) and cotrimoxazole prophylaxis. The risk factors for developing UTI were female, BMI ≥ 25 kg/m^2^, and higher degrees of immunologic risk including PRA ≥ 50% and the number of total HLA mismatches. Only recurrent UTI was associated with decreased allograft function. UTI in the first month after transplantation also significantly associated with decreased patient survival. Higher dosages of immunosuppressive medications were associated with the increased risk of having MDR organisms and recurrent UTI. Those who had ASB within the first 90 days had a higher incidence of UTI than recipients without ASB, regardless of the treatment.

Previous studies have shown that many recipient-, donor-, and transplantation-related risk factors contribute to the development of post-kidney transplant UTI [3,6,7]. In our study, female sex was the strongest risk factor which is probably explained by the anatomical differences of the female urinary tract compared with male recipients [28,29]. High immunologic risk also increased UTI risk, possibly related to the higher degree of immunosuppressive medications used [7]. BMI ≥ 25 kg/m^2^ has not been reported as a risk factor for UTI in kidney transplant recipients. However, previous studies in the general population demonstrated that being overweight or obese increased the risk of infection, including UTI, in children and adults [30,31,32]. This infection risk has been proposed to be related to chronic low-grade inflammation and immune dysfunction as a result of excessive adipose tissue-induced lymphatic system dysregulation [33,34,35]. Another possible explanation for increased UTI risk observed with high BMI in our study is the relatively higher dose of immunosuppressive medications given to these recipients, especially MPA and prednisolone, where routine therapeutic drug monitoring strategies are lacking. The Kidney Disease: Improving Global Outcomes (KDIGO) clinical practice guidelines on the evaluation and management of candidates for kidney transplantation suggests weight loss interventions be offered to candidates with obesity prior to transplantation, mainly because of the risk of surgical issues and delayed wound healing [36]. It still needs to be confirmed in future studies whether the incidence of post-transplantation UTI could be reduced by pre-transplant weight reduction.

The disparate effects of UTI on kidney allograft function which have been reported are possibly due to heterogeneity in immunosuppression protocols, which impacts kidney allograft function, as well as the mixed population of recipients with recurrent and non-recurrent UTI [37,38,39]. In our study where all recipients received the same immunosuppressive regimen, only recurrent UTI was associated with inferior allograft function, suggesting that repeated allograft damage may lead to interstitial fibrosis and tubular atrophy [40,41]. The risk factors for developing recurrent UTI in our study were tacrolimus dose and C_0_, which could be as a consequence of the long-term effects of immunosuppressive medications. This finding aligns with a previous study by Chen et al. who found tacrolimus C_0_ ≥ 8 ng/mL at first infection could independently predict repeat urinary tract and lung infections in kidney transplant recipients [42]. Recurrent UTI in our study was associated with poor allograft function. Therefore, in recipients with a history of UTI, clinicians should provide a thorough evaluation and assign appropriate interventions, in an effort to prevent further allograft damage. These interventions include, but are not limited to, identification of the cause of UTI such as anatomical abnormality detected via ultrasonography or abnormal voiding physiology detected via urodynamic study, the removal of foreign bodies in the urinary tract such as bladder catheters or ureteric stents, and retraining for proper voiding and cleaning technique [43,44]. Novel methods to prevent recurrent UTI such as probiotics, cranberries, and uropathogen vaccine seem promising, but additional clinical studies are needed, especially in a specific population such as kidney transplant recipients [45].

In addition to kidney allograft function, previous studies also showed conflicting results regarding the effect of UTI on patient and allograft survival [11,12,13,37,39,46,47,48]. These differences are likely to be caused by heterogeneity in the immunosuppressive medications used in different transplantation eras, the UTI prevention and treatment protocol in place at each transplant center, and the local antibiotic susceptibility profiles of the causative organisms. In our study, an overall UTI was not associated with patient or allograft survival. However, a secondary analysis revealed that UTI within the first month after transplantation was associated with decreased patient survival. This result is in line with a national retrospective study reported by O’Brien et al. that demonstrated higher mortality in veterans undergoing major surgery who had post-operative infection in the first 30 days after surgery [49]. Possible explanations could be both the direct and indirect links between early post-transplantation UTI and mortality. For the direct associations, the early post-operative period is a vulnerable period for kidney transplant recipients because the degree of immunosuppression is usually at its peak and the effects of induction therapy are still present. UTI that occurs during this period is more likely to be severe and associated with prolonged hospitalization, which negatively affects kidney transplant outcomes [50]. In addition, kidney transplant recipients who develop early UTI would not be able to return to work or resume a normal life, leading to financial problems, poor mental health, and lower quality of life, which can indirectly impact transplant outcomes [49].

The risk of having MDR organisms as a cause of UTI was found to relate with higher tacrolimus C_0_ and prednisolone daily dose. No studies have reported an association between the levels of immunosuppressive medications and the risk of drug-resistant organisms. We speculate that high degrees of immunosuppression impair surveillance immunity against uropathogens, allowing these organisms to proliferate and become the dominant pathogenic strain. It should be noted that tacrolimus not only affects adaptive immunity, but also innate immunity against urinary tract infection. Emal et al. demonstrated that tacrolimus can suppress the function of granulocytes and macrophages in an experimental mice UTI model, including the reduction of phagocytic activity, less cytolytic enzyme production, and deceased toll-like receptors expression [51]. Restoration of immune function by reducing the dose of immunosuppressants might enhance clearance of these drug-resistant organisms, for which the choices of antibiotics are limited [52].

ASB has been previously reported to increase the risk for developing UTI [53], and treatment of ASB has been proposed as another important cause of drug-resistant UTI. The latest guideline from the American Society of Transplantation Infectious Diseases Community of Practice notes that treating ASB within the first 2 months may have no benefit and may promote antimicrobial resistance [3]. Although study that investigates clinical benefits in treating ASB in the first 2–3 months after transplantation has always been lacking, two RCTs clearly showed that the treatment of ASB in kidney transplant recipients after 2 months post-transplantation did not translate to any clinical benefit but promoted the emergence of drug-resistant organisms [19,25]. Our study explored the potential benefit in treating ASB in the first 3 months; however, recipients with ASB progressed more frequently to UTI in the first transplant year regardless of treatment and were more likely to have UTI with MDR organisms, compared to recipients without ASB. Our results are consistent with a recent RCT that could not demonstrate a benefit of treating ASB in the first 2 months after kidney transplantation (and may even increase the incidence of UTI) [54]. We hypothesized that treatment of ASB may cause antibiotic selection pressure and increase the likelihood of infection with a pathogenic strain of the organisms, although not all would be MDR organisms by definition. This evidence suggests that treatment of ASB in the first 3 months should not be routinely recommended. Rather, individual recipients should receive a thorough evaluation of the source of bacteriuria, such as anatomical abnormality in the kidney/bladder, the presence of a foreign catheter/stent, or improper hygiene care. These sources should be removed or corrected as soon as possible, followed by a repeat urine culture, before a decision is made to treat with antibiotics. 

Interestingly, our study found that donor age was a risk of ASB, which has not been previously identified as a risk factor. We hypothesize that this association might relate to structural changes in the ageing kidney such as tubular dilatation and microcystic changes which could serve as a source for uropathogens [55,56]. These structural changes cannot be visualized via gross anatomical examination during donor nephrectomy and may promote ASB after transplantation [57]. If future studies confirm this finding, repeated screening for bacteriuria or using other detection methods, i.e., bacterial nuclease activity or multiplex recombinase polymerase amplification [58,59], in elderly donors might then be beneficial. 

There are several strengths of this study. The study population was homogenous in terms of immunosuppression regimens and UTI prophylaxis protocols and the timing of surgical device removal. The risks and association of UTI were comprehensively analyzed covering the important issues of allograft function, allograft survival, and patient survival, in the context of the modern era of immunosuppression. The risk factors and consequences of ASB were explored and the information regarding the early post-transplantation period has been added. However, in addition to the retrospective observational design, our study has some limitations. First, our center neither has a routine pre-transplantation screening protocol for bladder dysfunction or reflux nephropathy, nor is post-transplantation ultrasonography protocol beyond the first transplantation week. As a result, the prevalence of reflux nephropathy as a cause of ESRD could be underestimated. It should be mentioned that pyelonephritis in the native kidney is another cause of post-kidney transplantation UTI [60]. The incidence of post-transplantation kidney stone as a cause of UTI also could not be properly evaluated. Second, urine culture screening in the donor before deceased donor nephrectomy was not completely recorded, a factor which might contribute to development of ASB and post-transplant UTI. Third, we did not routinely screen for or treat ASB beyond 3 months post-transplantation, thus, outcomes of late ASB were not evaluated in our study. Finally, the timing of ureteric stents removal in our center occurs around 4 weeks after transplantation, which is later compared to some studies. Previous studies have demonstrated a benefit in reducing stent-related UTI if the ureteric stent is removed between 7 and 14 days, compared with beyond 4–6 weeks after transplantation [61,62]. However, the incidence of total UTI at 6 months post-transplantation was not different according to whether stents are removed early or late in one study [63]. Currently, we are trying to decrease the time to stent removal in our center to minimize the risk of stent-related UTI without increasing the risk of urologic complications.

In conclusion, bacterial UTI after kidney transplantation is associated with negative outcomes in terms of allograft function and patient survival. The risk factors for UTI included female sex, BMI ≥ 25 kg/m^2^, and higher degrees of immunologic risk. Our novel finding regarding UTI in overweight recipients suggests that these kidney transplant candidates should be advised to lose weight to minimize the risk of post-transplant UTI. Avoiding overimmunosuppression might be another strategy to prevent recurrent UTI or MDR organisms. ASB was associated with increased donor age. The benefit of screen-to-treat ASB within the first 3 months after transplantation could not be demonstrated and might be associated with an increased risk of drug-resistant organisms. However, an RCT is still needed to establish the benefits or risks of treating ASB within this early post-transplantation period.

## Figures and Tables

**Table 1:** Baseline patient characteristics.

Variables at the Time of Transplantation	Non-UTI Group (*n* = 219)	UTI Group (*n* = 129)	*p*-Value
Age, years (mean ± SD)	44.2 ± 12.1	44.9 ± 11.9	0.631
Female, *n* (%)	68 (31.1)	77 (59.7)	<0.001
BMI, kg/m^2^ (mean ± SD)	21.4 ± 3.2	21.9 ± 3.7	0.199
Mode of RRT, *n* (%)			0.104
Preemptive	11 (5.0)	3 (2.3)	
Hemodialysis	197 (90.0)	113 (87.6)	
Peritoneal dialysis	11 (5.0)	13 (10.1)	
Dialysis vintage, years (median, Q1–Q3)	2.9 (1.2–5.2)	3.8 (1.5–6.3)	0.099
Previous kidney transplantation, *n* (%)	9 (4.1)	7 (5.4)	0.571
Recipient diabetes mellitus, *n* (%)	29 (13.2)	22 (17.1)	0.331
Cause of kidney disease, *n* (%)			0.876
Glomerulonephritis	70 (32.0)	46 (35.7)	
Diabetic kidney disease	21 (9.5)	16 (12.4)	
Hypertensive nephrosclerosis	24 (11.0)	13 (10.1)	
Cystic kidney disease	10 (4.6)	5 (3.8)	
Urinary tract obstruction/reflux nephropathy	4 (1.8)	3 (2.3)	
Unknown	90 (41.1)	46 (35.7)	
Donor age, years (mean ± SD)	37.6 ± 12.1	38.0 ± 11.9	0.743
Deceased donor, *n* (%)	110 (50.5)	78 (61.4)	0.049
Donor serum Cr, mg/dL (mean ± SD)	1.55 ± 1.47	1.46 ± 1.16	0.593
HLA mismatch (mean ± SD)	2.7 ± 1.5	3.0 ± 1.6	0.084
PRA >50%, *n* (%)	15 (6.9)	24 (18.6)	0.001
ABO incompatible transplantation, *n* (%)	23 (10.5)	18 (14.0)	0.335
Total ischemic time >12 h, %	97 (45.1)	72 (56.7)	0.039
Delayed graft function, *n* (%)	51 (23.5)	35 (27.3)	0.426
Anti-thymocyte globulin induction, *n* (%)	25 (11.9)	22 (17.3)	0.164
Time to remove Foley catheter, days (median, Q1–Q3)	7 (7–7)	7 (7–7)	0.618
Time to remove drainage tube, days (median, Q1–Q3)	8 (8–9)	8 (8–9)	0.969
Time to remove double J stent, days (median, Q1–Q3)	26 (19–39)	28 (20–42)	0.345

BMI, body mass index; HLA, human leukocyte antigen; PRA, panel reactive antibody; UTI, urinary tract infection.

**Table 2:** Cox proportional hazard of baseline characteristics for developing UTI.

Variable	Univariable Analysis			Multivariable Analysis		
	HR	95%-CI	*p*-Value *	Adjusted HR	95%-CI	*p*-Value
Age (years)	1.00	0.99–1.02	0.667	-	-	-
Female (vs. male)	2.47	1.74–3.51	<0.001	2.25	1.56–3.24	<0.001
BMI ≥ 25 kg/m^2^	1.60	1.05–2.44	0.028	1.57	1.01–2.44	0.044
Hemodialysis (vs. preemptive)	2.07	0.66–6.50	0.215	-	-	-
Peritoneal dialysis (vs. preemptive)	3.77	1.07–13.24	0.038	2.92	0.81–10.55	0.103
Dialysis vintage (years)	1.05	0.99–1.10	0.093	0.99	0.93–1.07	0.879
Previous kidney transplantation	1.27	0.59–2.73	0.534	-	-	-
Recipient diabetes mellitus	1.29	0.81–2.04	0.281	-	-	-
Cystic kidney disease (vs. other causes of ESRD)	0.91	0.37–2.23	0.838	-	-	-
Obstructive/reflux nephropathy (vs. other causes of ESRD)	1.19	0.38–3.74	0.769	-	-	-
Deceased donor (vs. living donor)	1.47	1.03–2.10	0.035	1.00	0.46–2.19	0.991
Donor age (per 1 year increased)	1.00	0.99–1.02	0.697			
Donor serum creatinine (per 1 mg/dL)	0.97	0.84–1.11	0.649	-	-	-
HLA mismatch (per 1 mismatch)	1.12	0.99–1.26	0.067	1.14	1.01–1.29	0.041
PRA > 50% (vs. PRA ≤ 50%)	2.16	1.39–3.37	0.001	1.67	1.03–2.72	0.038
ABO incompatible transplantation	1.21	0.74–2.00	0.445	-	-	-
Total ischemic time >12 h	1.52	1.07–2.16	0.020	1.52	0.74–3.09	0.252
Delayed graft function	1.27	0.86–1.87	0.235	-	-	-
Anti-thymocyte globulin induction	1.28	0.81–2.03	0.294	-	-	-
Time to remove Foley catheter (per 1 day increased)	0.99	0.93–1.06	0.780	-	-	-
Time to remove drainage tube, days (per 1 day increased)	1.02	0.99–1.05	0.161	-	-	-
Time to remove double J stent (per 1 day increased)	1.01	0.99–1.01	0.152	-	-	-

* Variables with *p*-value less than 0.10 were included in the multivariable model. BMI, body mass index; ESRD, end-stage renal disease; HLA, human leukocyte antigen; HR, hazard ratio; PRA, panel reactive antibody.

**Table 3:** Urine culture result of the recipients in the UTI group.

UTI within the First Month (*n* = 63)		UTI in 2–12 Months (*n* = 35)		UTI after 12 Months (*n* = 31)	
Organism	%	Organism	%	Organism	%
*Escherichia coli*	63.4	*Escherichia coli*	54.3	*Escherichia coli*	54.8
*Klebsiella* spp.	22.2	*Klebsiella* spp.	14.3	*Klebsiella* spp.	19.3
*Enterococcus faecalis*	6.4	*Enterococcus faecalis*	8.6	*Enterococcus faecalis*	12.9
Negative culture	3.2	*Pseudomonas aeruginosa*	8.6	*Proteus* spp.	6.5
*Citrobacter* spp.	1.6	*Proteus* spp.	5.7	Negative culture	6.5
*Acinetobacter baumannii*	1.6	Negative culture	5.7		
*Serratia* spp.	1.6	*Citrobacter* spp.	2.8		

**Table 4:** Association between immunosuppressants at the time of (first) UTI diagnosis and MDR organisms or subsequent recurrent UTI via logistic regression analysis.

MDR Organisms	Univariable Analysis			Multivariable Analysis		
	OR	95%-CI	*p*-Value *	Adjusted OR	95%-CI	*p*-Value
Tacrolimus dose (per 1 mg/day increase)	1.20	1.04–1.39	0.012	1.12	0.95–1.33	0.181
Tacrolimus C_0_ (per 1 ng/mL increase)	1.17	1.02–1.35	0.030	1.20	1.02–1.42	0.032
MPA dose (per 250 mg/day of MMF or 180 mg/day of MPS increase)	1.43	1.05–1.94	0.024	1.16	0.78–1.72	0.459
Prednisolone dose (per 1 mg/day increase)	1.04	1.02–1.06	0.001	1.04	1.01–1.07	0.007
**Recurrent UTI**	**Univariable analysis**			**Multivariable analysis**		
	**OR**	**95%-CI**	*p*-value *	**aOR**	**95%-CI**	***p*-value**
Tacrolimus dose (per 1 mg/day increase)	1.20	1.04–1.38	0.012	1.26	1.07–1.50	0.007
Tacrolimus C_0_ (per 1 ng/mL increase)	1.26	1.09–1.47	0.002	1.28	1.10–1.50	0.002
MPA dose (per 250 mg/day of MMF or 180 mg/day of MPS increase)	1.14	0.84–1.53	0.405	-	-	-
Prednisolone dose (per 1 mg/day increase)	0.99	0.97–1.01	0.363	-	-	-

* Variables with *p*-value less than 0.10 were included in the multivariable model. C_0_, whole blood pre-dose concentration; MDR, multidrug-resistant; MMF, mycophenolate mofetil; MPS, (enteric-coated) mycophenolate sodium; OR, odds ratio; UTI, urinary tract infection.

**Table 5:** Univariable and multivariable logistic regression analysis of baseline characteristics for developing asymptomatic bacteriuria in the first 90 days after kidney transplantation.

Variable	Univariable Analysis			Multivariable Analysis		
	OR	95%-CI	*p*-Value *	Adjusted OR	95%-CI	*p*-Value
Age (years)	1.00	0.97–1.02	0.786	-	-	-
Female (vs. male)	4.15	2.04–8.43	<0.001	4.60	2.11–10.04	<0.001
BMI ≥ 25 kg/m^2^	0.99	0.42–2.35	0.979	-	-	-
Hemodialysis (vs. preemptive)	1.76	0.22–13.86	0.590	-	-	-
Peritoneal dialysis (vs. preemptive)	2.60	0.26–25.93	0.415	-	-	-
Dialysis vintage (years)	1.04	0.94–1.15	0.411	-	-	-
Previous kidney transplantation	1.04	0.23–4.76	0.957	-	-	-
Recipient diabetes mellitus	1.44	0.62–3.32	0.393	-	-	-
Cystic kidney disease (vs. other causes of ESRD)	0.51	0.07–3.97	0.519			
Obstructive/reflux nephropathy (vs. other causes of ESRD)	1.22	0.14–10.39	0.856			
Deceased donor (vs. living donor)	1.79	0.91–3.53	0.094	0.46	0.08–2.45	0.359
Donor age (per 1 year increase)	1.04	1.01–1.07	0.014	1.05	1.01–1.08	0.006
Donor serum creatinine (per 1 mg/dL)	1.06	0.83–1.34	0.656	-	-	-
HLA mismatch (per 1 mismatch)	1.07	0.86–1.32	0.562	-	-	-
PRA > 50% (vs. PRA ≤ 50%)	2.51	1.09–5.74	0.029	2.00	0.78–5.15	0.151
ABO incompatible transplantation	0.77	0.26–2.27	0.629	-	-	-
Total ischemic time >12 h	2.66	1.31–5.44	0.007	4.36	0.88–21.63	0.071
Delayed graft function	1.82	0.92–3.60	0.088	1.59	0.68–3.67	0.283
Anti-thymocyte globulin induction	1.07	0.42–2.69	0.892	-	-	-
Time to remove Foley catheter (per 1 day increased)	1.01	0.91–1.12	0.870	-	-	-
Time to remove drainage tube, days (per 1 day increased)	1.00	0.94–1.06	0.945	-	-	-
Time to remove double J stent (per 1 day increased)	1.00	0.98–1.02	0.865	-	-	-

* Variables with *p*-value less than 0.10 were included in the multivariable model. BMI, body mass index; ESRD, end-stage renal disease; HLA, human leukocyte antigen; OR, odds ratio; PRA, panel reactive antibody.

**Table 6:** Association between treated or untreated asymptomatic bacteriuria within the first 90 days after transplantation and MDR organisms in the first year after kidney transplantation. *p*-value from chi-square test was <0.001.

Asymptomatic Bacteriuria in the First 90 Days	No UTI in the First Year, *n* (%)	Non-MDR UTI in the First Year, *n* (%)	MDR UTI in the First Year, *n* (%)	Total
No asymptomatic bacteriuria	235 (76.8%)	31 (10.1%)	40 (13.1%)	306
Untreated asymptomatic bacteriuria	4 (40.0%)	2 (20.0%)	4 (40.0%)	10
Treated asymptomatic bacteriuria	11 (34.4%)	4 (12.5%)	17 (53.1%)	32

MDR, multidrug-resistant; UTI, urinary tract infection.

## Data Availability

The datasets generated and/or analyzed during the current study are not publicly available due to ethical considerations but are available from the corresponding author on reasonable request.

## Supplementary Materials

The following supporting information can be downloaded at: https://www.mdpi.com/article/10.3390/biomedicines10112984/s1, Table S1: Incidence of UTI and ASB according to BMI category; Table S2: Comparison between patients with simple cystitis, acute pyelonephritis, and asymptomatic bacteriuria that did not progress to UTI; Figure S1: Study flow diagram.
