# Supplementary material for: Bacterial Urinary Tract Infection and Early Asymptomatic Bacteriuria in Kidney Transplantation Still Negatively Affect Kidney Transplant Outcomes in the Era of Modern Immunosuppression and Cotrimoxazole Prophylaxis
Source: Biomedicines. 2022 Nov 20;10(11):2984. doi: 10.3390/biomedicines10112984 (PMC9687497; doi:10.3390/biomedicines10112984)

Bacterial Urinary Tract Infection and Early Asymptomatic Bacteriuria in Kidney Transplantation Still Negatively Affect Kidney Transplant Outcomes in the Era of Modern Immunosuppression and Cotrimoxazole Prophylaxis

Chayanan Santithanmakorn,[1] Jakapat Vanichanan,[2] Natavudh Townamchai,[3,4,5] Kamonwan Jutivorakool,[2] Salin Wattanatorn,[3] Methee Sutherasan,[4,6] Julin Opanuruk,[6] Stephen J. Kerr,[7,8,9] Kearkiat Praditpornsilpa,[3] Yingyos Avihingsanon,[3,4,5] Suwasin Udomkarnjananun[3,4,5]

[1]Department of Medicine, Faculty of Medicine, Chulalongkorn University and King Chulalongkorn Memorial Hospital, Bangkok, Thailand

[2]Division of Infectious Diseases, Department of Medicine, Faculty of Medicine, Chulalongkorn University and King Chulalongkorn Memorial Hospital, Bangkok, Thailand

[3]Division of Nephrology, Department of Medicine, Faculty of Medicine, Chulalongkorn University and King Chulalongkorn Memorial Hospital, Bangkok, Thailand

[4]Excellence Center for Organ Transplantation (ECOT), King Chulalongkorn Memorial Hospital, Thai Red Cross Society, Bangkok, Thailand

[5]Renal Immunology and Transplantation Research Unit, Faculty of Medicine, Chulalongkorn University, Bangkok, Thailand

[6]Department of Surgery, Faculty of Medicine, Chulalongkorn University and King Chulalongkorn Memorial Hospital, Bangkok, Thailand

[7]Biostatistics Excellence Centre, Research Affairs, Faculty of Medicine, Chulalongkorn University, Bangkok, Thailand.

[8]HIV-NAT, Thai Red Cross AIDS Research Centre, Bangkok, Thailand

[9]The Kirby Institute, University of New South Wales, Sydney 2052, Australia.

Address for Correspondence: Suwasin Udomkarnjananun, Division of Nephrology, Department of Medicine, Faculty of Medicine, Chulalongkorn University and King Chulalongkorn Memorial Hospital, 1873, Rama IV Road, Bangkok, 10330, Thailand.

E-mail: suwasin.u@gmail.com

Supplementary Table S1: Incidence of UTI and ASB according to BMI category.

BMI (kg/m^2)	No UTI or ASB	ASB	Simple cystitis	Acute pyelonephritis (complicated UTI)
< 25	189 (86%)	35 (83%)	8 (73%)	93 (79%)
25-29.9	27 (12%)	5 (12%)	2 (18%)	23 (19%)
≥ 30	3 (2%)	2 (5%)	1 (9%)	2 (2%)
Total	219 (100%)	42 (100%)	11 (100%)	118 (100%)

ASB; asymptomatic bacteriuria; BMI: body mass index; UTI; urinary tract infection

Supplementary Table S2: Comparison between patients with simple cystitis, acute pyelonephritis, and asymptomatic bacteriuria that did not progress to UTI.

**Variables at the time of transplantation**	**ASB not progress to UTI (n = 9)**	**Simple cystits (n = 11)**	**Acute pyelonephritis (n = 118)**	**p-value**
Age, years (mean±SD)	52±15	45±11	45±12	0.218
Female, n (%)	7 (78)	9 (82)	68 (58)	0.163
BMI, kg/m² (mean±SD)	21.9±3.4	22.6±4.7	21.8±3.6	0.816
Mode of RRT, n (%)				0.493
Preemptive	1 (11)	0 (0)	3 (3)	
Hemodialysis	8 (89)	10 (91)	103 (87)	
Peritoneal dialysis	0 (0)	1 (9)	12 (10)	
Dialysis vintage, years (median, Q1-Q3)	3.1 (0.5-3.8)	5.0 (1.8-7.3)	3.6 (1.5-6.3)	0.343
Previous kidney transplantation, n (%)	1 (11)	1 (9)	6 (5)	0.672
Recipient diabetes mellitus, n (%)	4 (44)	2 (18)	20 (17)	0.126
Cause of kidney disease, n (%)				0.821
Glomerulonephritis	2 (22)	5 (45)	41 (35)	
Diabetic kidney disease	3 (33)	1 (9)	15 (13)	
Hypertensive nephrosclerosis	1 (11)	2 (18)	11 (9)	
Cystic kidney disease	0 (0)	0 (0)	5 (4)	
Urinary tract obstruction/reflux nephropathy	0 (0)	0 (0)	3 (3)	
Unknown	3 (33)	3 (27)	43 (36)	
Donor age, years (mean±SD)	43±7	36±14	38±12	0.429
Deceased donor, n (%)	3 (33)	6 (55)	72 (61)	0.239
Donor serum Cr, mg/dL (mean±SD)	1.4±1.0	1.5±0.3	1.5±1.2	0.998
HLA mismatch (mean±SD)	2.5±0.7	3.0±1.5	2.9±1.6	0.729
PRA >50%, n (%)	2 (22)	3 (27)	21 (18)	0.718
ABO incompatible transplantation, n (%)	1 (11)	0 (0)	18 (15)	0.363
Total ischemic time >12 hours, %	4 (44)	6 (55)	66 (56)	0.469
Delayed graft function, n (%)	2 (22)	3 (27)	32 (27)	0.927
Anti-thymocyte globulin induction, n (%)	2 (22)	3 (27)	19 (16)	0.473
Time to remove Foley catheter, days (median, Q1-Q3)	7 (7-7)	7 (7-7)	7 (7-7)	0.725
Time to remove drainage tube, days (median, Q1-Q3)	8 (8-8)	8 (7-8)	8 (8-9)	0.684
Time to remove double J stent, days (median, Q1-Q3)	23 (22-45)	30 (17-39)	28 (21-42)	0.729
eGFR at 1 year, mL/min/1.73 m² (mean±SD)	56±21	54±21	59±20	0.778
eGFR at 3 year, mL/min/1.73 m² (mean±SD)	56±18	56±22	58±23	0.939
Graft survival function at 1 year	0.89	1.00	0.97	-
Graft survival function at 5 year	0.89	0.91	0.90	-
Patient survival function at 1 year	0.89	1.00	0.97	-
Patient survival function at 5 year	0.89	0.90	0.91	-

ASB, asymptomatic bacteriuria; BMI, body mass index, HLA; human leukocyte antigen, PRA; panel reactive antibody, UTI; urinary tract infection

Supplementary Figure S1: Study flow diagram

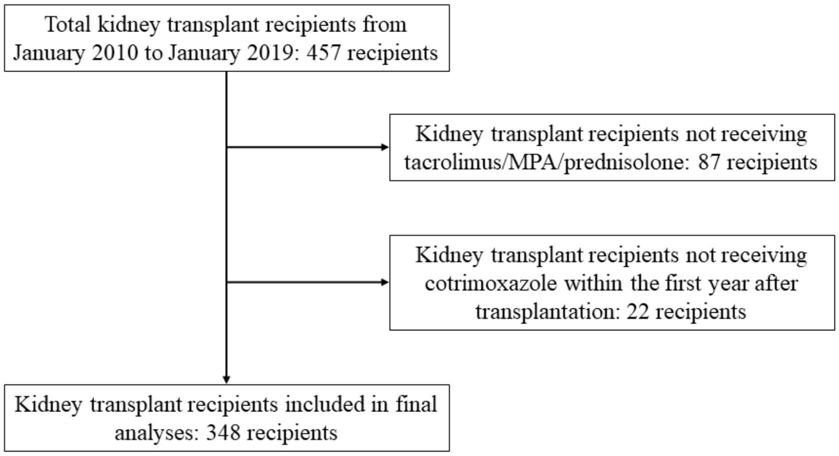

Supplement: Supplementary file 1 [file biomedicines-10-02984-s001.zip › Supplementary UTI revised.pdf]
